# Transcriptome sequencing and analysis of *Plasmodium gallinaceum* reveals polymorphisms and selection on the apical membrane antigen-1

**DOI:** 10.1186/1475-2875-13-382

**Published:** 2014-09-26

**Authors:** Elvin J Lauron, Khouanchy S Oakgrove, Lisa A Tell, Kevin Biskar, Scott W Roy, Ravinder NM Sehgal

**Affiliations:** Department of Biology, San Francisco State University, San Francisco, CA 94132 USA; Department of Medicine and Epidemiology, School of Veterinary Medicine, University of California, Davis, CA 95616 USA

**Keywords:** Avian malaria, Transcriptome, Apical membrane antigen I, Synonymous, Non-synonymous, Polymorphism

## Abstract

**Background:**

*Plasmodium* erythrocyte invasion genes play a key role in malaria parasite transmission, host-specificity and immuno-evasion. However, the evolution of the genes responsible remains understudied. Investigating these genes in avian malaria parasites, where diversity is particularly high, offers new insights into the processes that confer malaria pathogenesis. These parasites can pose a significant threat to birds and since birds play crucial ecological roles they serve as important models for disease dynamics. Comprehensive knowledge of the genetic factors involved in avian malaria parasite invasion is lacking and has been hampered by difficulties in obtaining nuclear data from avian malaria parasites. Thus the first Illumina-based *de novo* transcriptome sequencing and analysis of the chicken parasite *Plasmodium gallinaceum* was performed to assess the evolution of essential *Plasmodium* genes.

**Methods:**

White leghorn chickens were inoculated intravenously with erythrocytes containing *P. gallinaceum*. cDNA libraries were prepared from RNA extracts collected from infected chick blood and sequencing was run on the HiSeq2000 platform. Orthologues identified by transcriptome sequencing were characterized using phylogenetic, *ab initio* protein modelling and comparative and population-based methods.

**Results:**

Analysis of the transcriptome identified several orthologues required for intra-erythrocytic survival and erythrocyte invasion, including the rhoptry neck protein 2 (RON2) and the apical membrane antigen-1 (AMA-1). *Ama*-*1* of avian malaria parasites exhibits high levels of genetic diversity and evolves under positive diversifying selection, ostensibly due to protective host immune responses.

**Conclusion:**

Erythrocyte invasion by *Plasmodium* parasites require AMA-1 and RON2 interactions. AMA-1 and RON2 of *P. gallinaceum* are evolutionarily and structurally conserved, suggesting that these proteins may play essential roles for avian malaria parasites to invade host erythrocytes. In addition, host-driven selection presumably results in the high levels of genetic variation found in *ama*-1 of avian *Plasmodium* species. These findings have implications for investigating avian malaria epidemiology and population dynamics. Moreover, this work highlights the *P. gallinaceum* transcriptome as an important public resource for investigating the diversity and evolution of essential *Plasmodium* genes.

**Electronic supplementary material:**

The online version of this article (doi:10.1186/1475-2875-13-382) contains supplementary material, which is available to authorized users.

## Background

Immuno-evasion is likely a major factor that influences the evolution of *Plasmodium* parasites, the causative agent of malaria [1]. Parasites with antigenic diversity may have frequency-dependent advantages and as a result evolve under strong positive/diversifying selection [2]. The genetic diversity maintained by positive selection in target malaria antigens poses a major problem in the development of effective malaria vaccines [3, 4]. In general, most genes that encode antigens of *Plasmodium* parasites are highly polymorphic and encode proteins that are important targets for host protective antibody responses [5]. Indeed, polymorphisms in the circumsporozoite protein, a cell surface protein required for sporozoites to attach and invade target cells, appear to be maintained by selective pressures exerted via host protective immune responses [6, 7]. Evidence for positive selection has also been reported for the *Plasmodium* surface proteins DBP (Duffy-binding protein), EBA-175 (erythrocyte-binding antigen 175) and a large number of other antigens [8].

Polymorphisms maintained by selective pressures within erythrocyte-binding ligands may alter the host receptor-specificity of *Plasmodium* parasites [3, 9, 10]. In *Plasmodium* parasites that infect multiple host species through host-switching events, e.g., avian malaria species, such polymorphisms may contribute to a broad host-specificity range [11]. The host-specificity of avian malaria parasites is diverse: some parasites can infect hosts from multiple families or even multiple orders; others are restricted to a single avian family or even species [12–14]. Therefore, avian *Plasmodium* parasites provide an exceptional model for studying host specificity and host-parasite co-evolutionary dynamics in natural populations [15, 16].

Host-parasite co-evolutionary relationships are thought to maintain genetic diversity in both host and parasite populations [17]. Indeed, there is evidence for parasite-driven diversifying selection in avian hosts [18–20]. However, little is known regarding host-driven selection in avian *Plasmodium* parasites. This is largely due to the difficulties of identifying and obtaining data on nuclear genes: since erythrocytes are nucleated in bird hosts, it is hard to isolate parasite DNA/RNA from the much more abundant host material [21]. Therefore, *Plasmodium gallinaceum*, a parasite of the domestic chicken (*Gallus gallus*), was chosen to perform transcriptome sequencing and analysis in this study since it is relatively easy to propagate in chickens and generating high parasitemia is readily achieved with this strain.

*Plasmodium gallinaceum* has been an important model for understanding cellular biological mechanisms involved in malaria parasite transmission [22–24], and can yield insight applicable to *Plasmodium falciparum* [25], as *P. falciparum* shares high similarity with the genome of *P. gallinaceum.* This is supported by phylogenetic evidence [26–31] and biochemical data that functionally confirm the evolutionary relationships [25]. Here, the goal was to identify orthologues of essential and well-characterized *P. falciparum* genes from the *P. gallinaceum* transcriptome; some of these include the long chain fatty acid elongation enzyme (*ELO3*), LCCL domain-containing protein (*CCp2*), and serine hydroxymethyltransferase (*SHMT*).

*SHMT* is highly upregulated throughout the intra-erythrocytic development stages [32] and plays an indispensable role in the *de novo* pyrimidine biosynthesis pathway in *Plasmodium* parasites; the essentiality of *SHMT* has been confirmed through *SHMT*-knockout parasites [33]. *ELO3* and *CCp2* also play essential roles during intra-erythrocytic development and are specifically required for *Plasmodium* gametocytogenesis, as demonstrated by transposon-mediated insertional mutagenesis [34]. In addition, two orthologues that are essential for erythrocytic invasion, AMA-1 (a major malaria vaccine candidate) and RON2 (the AMA-1 receptor) were characterized in this study.

*Plasmodium* invasion of erythrocytes can be blocked by antibody-mediated inhibition of AMA-1-RON2 interactions [35–37], and the vaccine potential of AMA-1 has been well demonstrated in various animal models [38–40]. In spite of these promising results, different isolates of the same species exhibit polymorphisms in *ama*-*1* that may allow parasites to avoid inhibitory effects of natural anti-AMA1 antibodies produced by host protective immune responses [41]. Moreover, natural immune responses to AMA-1 have also revealed polymorphic B and T cell epitopes within AMA-1 that are maintained by positive selection [42, 43]. Given that AMA-1 is a highly polymorphic antigen that is unique to apicomplexan parasites [44, 45], the level of diversity and selection on avian *Plasmodium ama*-*1* was evaluated. The results of this study have implications for studying erythrocyte invasion, host immune responses and the population genetics and epidemiology of avian malaria parasites.

## Methods

### Infection of chickens

A total of seven White Leghorn chickens were hatched at the animal facility of the University of California, Davis, and were kept in cages with water and feed. After six days, six chicks were inoculated intravenously with erythrocytes containing *P. gallinaceum.* One chick was inoculated with saline solution as a negative control. Blood samples were obtained from the jugular vein seven days post infection and subsequently every two to three days. Blood samples were stored in TRIzol® RNA Isolation Reagent (Life Technologies, USA) and were immediately flash frozen in an ethanol-dry ice bath, or used for blood smear examination. Blood smears were stained with Giemsa, and the infection status was verified by microscopy and PCR amplification of the *cytochrome b* gene [46].

### Generation of cDNA libraries, sequencing and data analysis of the *Plasmodium gallinaceum*blood stages

Total RNA was prepared directly from the frozen samples of parasitized erythrocytes. RNA was extracted using Phase Lock Gel and ethanol precipitation methods [47]. The RNA quality was checked on the Bioanalyzer 2100 (Agilent Technologies Inc., USA). cDNA libraries were prepared from RNA extracts and sequenced at the qb3 Genomics Sequencing Laboratory at the University of California, Berkeley, USA as follows: rRNA was depleted from RNA extracts using Ribo-Zero™ (Epicentre, USA) prior to generating cDNA libraries using TruSeq™ (Illumina Inc, USA). Sequencing was run in one lane as paired-end reads of 100 base pairs (bp) on the HiSeq2000 platform. The quality of all Illumina reads was assessed with FastQC [48]. Overall, the sequence reads were of good quality (average quality score of 38 per read). Seventy-five per cent of the sequence reads had a quality score ≥ 30. Thus, no quality trimming was required nor performed, so as to minimize loss of the dataset. Blat/Bowtie [49, 50] query of the Illumina reads against the *G. gallus* (chicken) genome was run to remove chicken sequences. Adapters were removed and the remaining paired-end reads were used for the *de novo* reconstruction of the *P. gallinaceum* transcriptome using Trinity [51]. To identify *P. gallinaceum* protein-coding transcripts involved in erythrocyte invasion, intra-erythrocytic survival and gametocytogenesis, Tblastn of the *P. gallinaceum* transcriptome against the *P. falciparum* transcriptome was performed using Geneious 7.0.4. An E-value cut-off of 1e-10 was chosen for identifying putative orthologues. The *P. falciparum* transcriptome was downloaded from PlasmoDB [52].

### Sample collection

*Plasmodium lucens* isolates used in this study came from blood samples collected from a single species, the Olive Sunbird (*Cyanomitra olivacea*), in Cameroon during the period 2005 to 2007 [46]. *Plasmodium globularis* was isolated from blood samples collected from the Yellow-whiskered Greenbul (*Andropadus latirostris*) in Ghana, 2007 [53]. *Plasmodium megaglobularis* isolates came from blood samples collected from the Olive-bellied Sunbird (*Cinnyris chloropygius*) in Cameroon during 1990 [53]. *Plasmodium* lineage spp. PV16 isolates were from blood samples collected from the Olive Sunbird in Cameroon during 2005 [14]. *Plasmodium homopolare* isolates were collected from various birds in China Creek County Park, California, USA (Additional file 1) during 2011 to 2013 [54]. All birds were caught with mist-nets and banded. Blood samples were collected from the brachial vein and samples were stored in lysis buffer (10 mM Tris-HCL pH 8.0, 100 mM EDTA, 2% SDS).

### PCR amplification and DNA sequencing

DNA was extracted from whole blood following a DNeasy kit protocol (Qiagen, USA). Identification of avian *Plasmodium* species was based on PCR assays and sequences of the *cytochrome b* gene [55]. The *P. gallinaceum ama-1* coding sequence was identified using the *P. gallinaceum* RNA-seq data, and *ama-1* domain I primers were designed based on conserved regions among *P. gallinaceum* and other mammalian *Plasmodium* species. A nested PCR was used to amplify the hypervariable domain I region of *ama-1* corresponding to 444-906 bp or 271-732 bp according to *P. falciparum ama*-*1* or *P. gallinaceum ama-1*, respectively. The following primers were used for the first round of amplification: *Pg*_*AMA1*F1 (GATTTAGGTGAAGATGCAGAAGT) and *Pg*_*AMA1*R1 (TTAATTAAACATGTTGGTTTTACAT). The amplification conditions were as follows, first, 4 min at 94°C, followed by 20 cycles with 0.5 min of denaturation at 94°C, annealing at 50°C for 1 min, and elongation at 72°C for 1.2 min. After 20 cycles, a final elongation step at 72°C for 5 min was carried out. The amplified products of 785 bp were used for the second round of amplification with the following primers: *Pg*_*AMA1*F2 (ATGTCCAGTTTTTGGAAAAGGTAT) and *Pg*_*AMA1*R2 (CCATCAACCCATAAT CCAAATTT). The second round amplification conditions were as follows first, 1 min at 94°C, followed by 40 cycles with 0.5 min of denaturation at 94°C, annealing at 53°C for 1 min, and elongation at 72°C for 0.7 min. After 40 cycles, a final elongation step at 72°C for 5 min was carried out. The amplified products of 500 bp were run out on a 1.8% agarose gel using 1 × TBE, and visualized by ethidium bromide staining under ultraviolet light. Resulting amplicons were purified using ExoSap (following manufacture’s instructions, USB Corp, USA) and sequenced by ElimBio (Hayward, USA), see Additional file 2 and Additional file 3 for accession numbers. Several attempts to amplify domain II and III were unsuccessful, which may have been due to the low GC content in these regions and the difficulty in designing highly specific primers.

### Phylogenetic analyses

DNA sequences were analysed using Geneious v7.0.4 created by Biomatters. Sequences were aligned using MUSCLE with Seaview software [56]. DNA sequences were translated and adjusted in Mesquite v2.75 [57]. Phylogenetic relationships were inferred using maximum likelihood (ML), as implemented in RAxML [58]. For ML, Modeltest v3.7 [59] was used to determine the most appropriate nucleotide substitution model based on the Akaike Information Criterion (AIC) [60]. ML methods for the *ama*-*1* and *SHMT* genes were implemented using the GTR + I + G model that permits rate variations in all six base substitution types for unequal base composition, invariable sites and among site rate variation. ML methods for the *RON2*, *ELO3*, *CCp2* genes were implemented using the GTR + G model. A thorough ML search was performed along with 10,000 bootstrap inferences.

### Protein structure modelling

Three-dimensional (3D) models representing tertiary protein structures of AMA-1, domain I of AMA-1 alone, and RON2 was generated using an *ab initio* approach with the iterative implementation of the threading assembly refinement (I-TASSER) method [61, 62]. The accuracy of the 3D models was assessed based on the confidence (C) score and the template modelling (TM) score. The quality of the top-ranked 3D models, measured by LGscore and MaxSub values, was further assessed using protein quality predictor ProQ [63]. 3D models with an LGscore greater than 2.5 are considered very good models, where as values above 4 indicate extremely good models. 3D models with MaxSub values above 0.1 are considered fairly good models, whereas values above 0.5 indicate very good models. Secondary structures of AMA-1 or domain I of AMA-1 alone and RON2 were determined by PSIPRED v3.3 [64].

### Statistical analyses of genetic diversity

A total of 51 *P. lucens ama*-*1* sequences consisting of 12 different haplotypes were compared with 49 *P. falciparum ama*-*1* sequences. The McDonald-Kreitman test [65] was performed on domain I of *ama-1* to determine whether this region is evolving under selection. The *d*_N_/*d*_S_ (non-synonymous substitutions per non-synonymous sites divided by synonymous substitutions per synonymous sites) ratio was evaluated using a sliding window method to investigate selection across the region, as implemented in DNAsp v5.10 [66]. Significant differences between *d*_N_ and *d*_S_ were evaluated for the entire region of domain I and for regions with high *d*_N_/*d*_S_ ratios using the Nei and Gojobori method with the Jukes and Cantor correction, and a one-tailed Z-test with 1,000 bootstrap pseudosamples, as implemented in MEGA v5.2.2 [67]. Between-species divergence (*K*) using Jukes and Cantor correction was calculated with MEGA (see Additional file 4 and Additional file 5). The nucleotide diversity (π) across domain I of *P. lucens ama-1* was also evaluated using a sliding window method.

Tajima’s test was performed to determine if sequences departed significantly from neutral variation patterns. With Tajima’s test, departure from neutrality is measured by differences between π and the nucleotide diversity expected under neutrality (θ). π is expected to increase above that of θ as a result of a rare allele being selected and maintained at intermediate frequencies under positive selection. Thus, a positive test statistic (*D*) value under positive diversifying selection is expected [68, 69]. Fu and Li’s test was also performed using *P. gallinaceum* as outgroup to determine whether mutations are selectively neutral. Similarly, a positive value of D* and F* under positive diversifying selection is expected. When comparing estimates of θ based on singleton sites to that derived from the D* or F* index, an excess of intermediate frequency polymorphisms and lower number of singleton sites makes the statistics values positive [69, 70]. To check for clustering, a metric multidimensional scaling analysis was performed in R using the bios2mds package.

## Results

### Transcriptome sequencing

A total of 100 Gb comprising of 220 M 100 nucleotide (nt) paired-end sequencing read was obtained. Sixty-three percent of the total sequence reads obtained were removed after running a Bowtie query against the *G. gallus* genome. The remaining 82 M sequence-read pairs were assembled *de novo* using Trinity. Long open reading frames (ORFs) within the assembled transcriptome were identified. These putative coding sequences (CDS) were compared to the *P. falciparum* (isolate 3D7) transcriptome. Eighty-one per cent of *P. falciparum* CDS sequences had a significant BLAST hit within the *P. gallinaceum* transcriptome, suggesting that the *P. gallinaceum* transcriptome obtained was fairly complete. The size distribution for the CDS that showed homology to *P. falciparum* CDS is shown in Additional file 6.

### Identification and phylogenetic analysis of genes essential for intra-erythrocytic stage survival, and gametocytogenesis

A full-length cDNA sequence encoding an orthologue of SHMT in *P. gallinaceum,* with an 84% amino acid sequence identity in a pairwise comparison to *P. falciparum* SHMT, was identified*.* The translated *SHMT* sequences of all analysed *Plasmodium* species resulted in proteins with identical lengths of 442 amino acids. Twelve cysteines were present in *P. gallinaceum* SHMT, eight of which were conserved in position.

**Figure 1:**
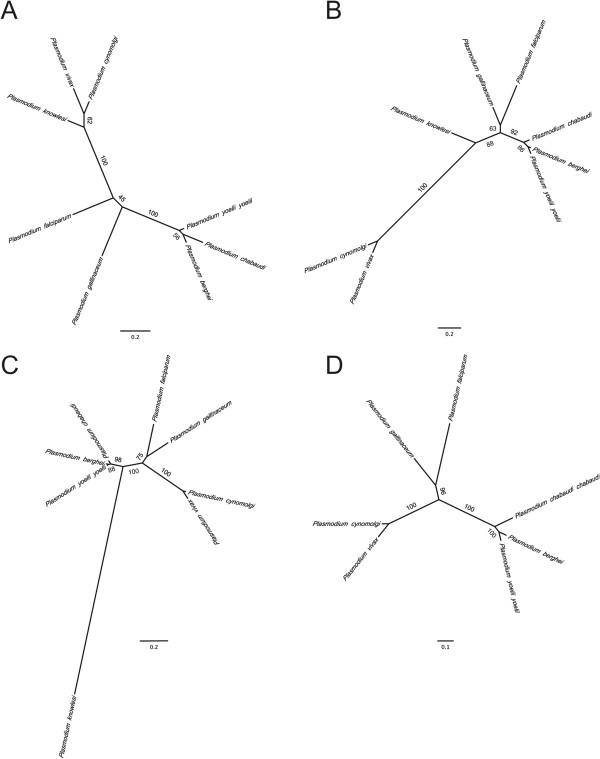
**Phylogeny of** ***Plasmodium*** **parasites based on A) 1,329 bp of the serine hydroxymethyltransferase (** ***SHMT*** **) gene, B) 1920 bp of the long chain fatty acid elongation enzyme (** ***ELO3*** **) gene, C) 4854 bp of the LCCL domain-containing protein (** ***CCp2*** **) gene, and D) 7563 bp of the Rhoptry neck protein 2 (** ***RON2*** **) gene. Numbers on the branches refer to bootstrap values obtained with 10,000 replicates.** For accession numbers see Additional file 2.

In addition, *ELO3* and *CCp2* were also identified as orthologues in *P. gallinaceum*. The full-length cDNA sequence was obtained for *ELO3* and a partial cDNA sequence was obtained for *CCp2*. The 5’ exon sequence of *CCp2* was missing approximately 650 bp. The resulting coding sequences of *ELO3* and *CCp2* are 1,920 and 4,201 bp, respectively. DNA sequences were translated and aligned; the resulting amino acid sequences showed 79 and 74% identity in pairwise comparisons to *P. falciparum* ELO3 and CCp2, respectively. The *P. gallinaceum* CCp2 amino acid sequence (1,348 amino acids) that was analysed contains 17 cysteines, all of which were conserved between mammalian *Plasmodium* species. The translated *P. gallinaceum ELO3* full-length transcript sequence resulted in a protein of 521 amino acids and was approximately 121 amino acids shorter than the *P. falciparum* ELO3 protein. Phylogenetic analyses suggested that these essential orthologues in *P. gallinaceum* are most similar to *P. falciparum* (as compared to other mammalian *Plasmodium* species) (Figure 1A-C). The high degree of conservation suggests that these orthologues may also play important roles during the intra-erythrocytic stages of *P. gallinaceum*.

### Identification and phylogenetic analysis of *ama-1*and the *ama-1*receptor *RON2*in *Plasmodium gallinaceum*

**Figure 2:**
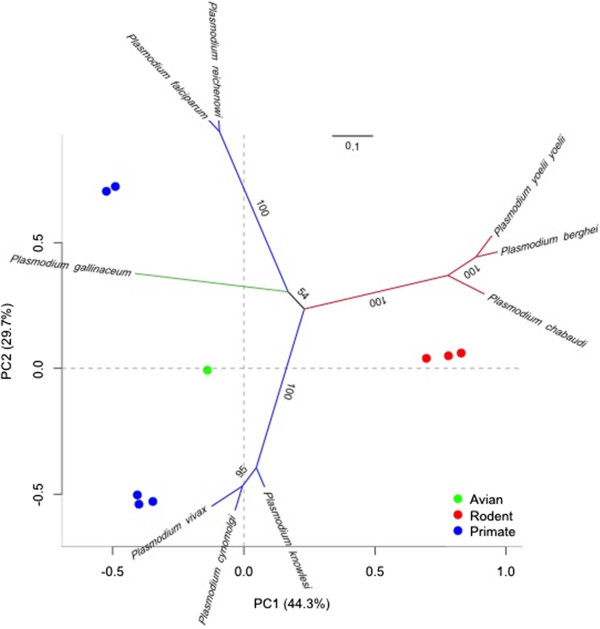
**Phylogeny and metric multidimensional scaling of** ***Plasmodium*** **parasites using** ***ama*** **-** ***1*** **.** A matrix of distances (p-distance) were generated from aligned *ama*-*1* sequences. The distance matrix was used to calculate principal components and is represented by metric multidimensional scaling (MDS). For graphical purposes, the phylogeny was superimposed onto a PCA plot to visualize sequence space.

The coding region of *P. gallinaceum ama*-*1* was 1,692 bp with an A + T content (72%) greater than *P. falciparum* (around 70%). The translated protein sequence was 556 amino acids long, shared 55% amino acid identity to *P. falciparum ama-1*, and contains 17 cysteines. All 16 cysteines within the three cysteine-rich domains of AMA-1 were conserved in number and position when the aligned protein sequences of individual domains were analysed, with the exception of domain III. Domain III contains six cysteine residues [71, 72], which were present in both *P. gallinaceum* and *P. falciparum ama*-*1*. However, domain III of *P. gallinaceum* AMA-1 contained five amino acid deletions between positions 465-471 (relative to *P. falciparum* AMA-1). Therefore, the position of cysteines and the number of amino acids in domain III varied slightly between *P. gallinaceum* and *P. falciparum*. Phylogenetic analysis of *ama*-*1* revealed *P. gallinaceum ama*-*1* as significantly divergent from all mammalian *Plasmodium* species analysed (Figure 2). A metric multidimensional scaling (MDS) analysis was performed to complement the phylogeny and to visualize the evolutionary trajectories of *ama*-*1* on a low dimensional space. Principal component analysis (PCA) plots showed clustering consistent with that of the *ama*-*1* phylogeny (Figure 2). Phylogenetic and metric multidimensional scaling analysis of all *ama*-*1* sequences compared in this study placed avian *Plasmodium* parasites into a strongly supported monophyletic clade and cluster (Additional file 7).

**Figure 3:**
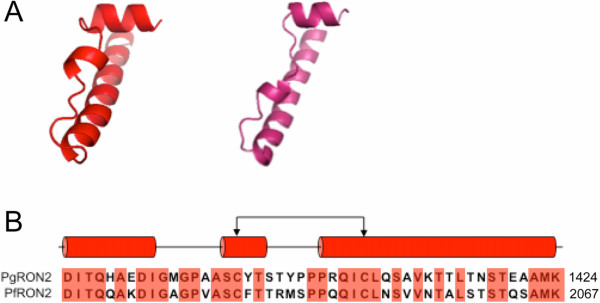
**Three-dimensional models of the avian** ***Plasmodium*** **RON2 protein. A)** Stereo views of the C-terminus of *P. gallinaceum* RON2 (left) and *P. falciparum* RON2 (right). **B)** Sequence alignment of *P. gallinaceum* and *P. falciparum* RON2 are shown with the secondary structure of the corresponding amino acid regions above the alignment. Helices are colored red. The line with connecting arrows indicates disulfide bonds. Conserved amino acids are highlighted in red.

In addition to identifying AMA-1 in *P. gallinaceum*, a full-length cDNA sequence encoding a version of *P. gallinaceum* RON2 was identified. *P. gallinaceum* RON2 was truncated at the N terminus by approximately 620 amino acids, in comparison to the translated full-length cDNA sequence of *P. falciparum* RON2. The coding sequence of *P. gallinaceum RON2* was 4,641 bp and encodes a protein of 1545 amino acids with 71% identity to *P. falciparum* RON2. Two conserved cysteines that are required for RON2 to bind the AMA-1 pocket [73] were conserved in *P. gallinaceum* RON2 (Figure 3). A phylogenetic analyses of RON2 groups *P. gallinaceum* with *P. falciparum* (Figure 1D).

### *ama-1*polymorphisms in avian *Plasmodium*field isolates

**Figure 4:**
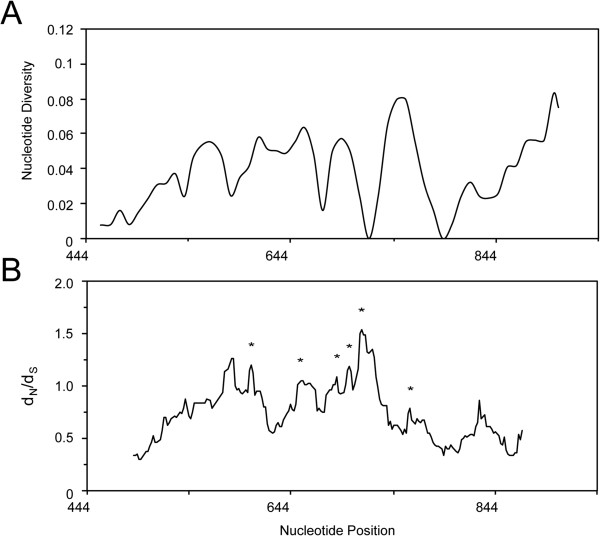
**Sliding window plot of A) π and B)** ***d***_**N**_ **/** ***d***_**S**_ **for domain I of** ***Plasmodium lucens ama*** **-** ***1*** **.** Nucleotide positions are relative to the *P. falciparum* 3D7 line sequence. The window length is 30 bp with a step size of 9 bp for the sliding window analysis of π. The window length is 90 bp with a step size of 3 bp for the sliding window analysis of *d*_N_/*d*_S_. Asterisks indicate regions with a significant excess of non-synonymous substitutions.

The entire *ama*-*1* domain I region consisting of 468 bp was sequenced and analysed from a total of 51 *P. lucens* isolates collected from African rainforest birds. A sliding window analysis of π using a window of 30 bp moved in steps of nine sites reveals polymorphisms across the entire region of domain I (Figure 4A). The region 787-816 bp appears to be the most polymorphic region in domain I of *P. lucens ama*-*1*. This region corresponds to the naturally immunogenic T cell epitope located within residues 259-271 of *P. falciparum* AMA-1, which was also reported to be polymorphic [42]. Three-hundred and eighty-seven monomorphic sites and 81 polymorphic sites were detected in domain I of *P. lucens ama*-*1*. Three polymorphic sites at positions 116, 138 and 205 exhibited three different nucleotides, whereas the remaining sites had only two. A total of 77 mutations were detected, 27 of which were synonymous and 50 of which were non-synonymous. π for all 51 sequences analysed was 0.043237 ± 0.005210 SD, which is more polymorphic than previously reported π (0.01361-0.01764) for *P. falciparum* isolates [42, 74].

**Figure 5:**
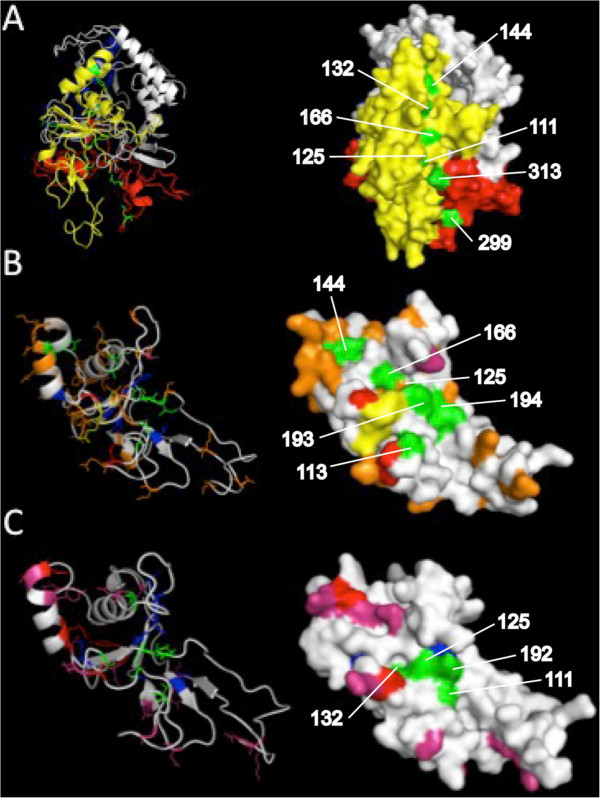
**Three-dimensional models of the avian** ***Plasmodium*** **AMA-1 protein.** The *ab initio*-generated models are based on the **A)** 556 aa sequence of *P. gallinaceum* AMA-1, **B)** 155 aa residues in AMA-1 domain I of *P. lucens*, and **C)** 152 aa residues in AMA-1 domain I of *P. falciparum*. Both stereo (left) and surface (right) views are shown. Domain I, II and III of *P. gallinaceum* AMA-1 are coloured yellow, red and blue, respectively. Conserved hydrophobic residues that line the putative hydrophobic trough are labelled and highlighted in green. *Plasmodium lucens* and *P. falciparum* AMA-1 models are coloured as follows: conserved hydrophobic residues are shown in blue, highly polymorphic residues are shown in red, high-frequency dimorphisms are shown in pink, low-frequency dimorphisms are shown in orange. The observed amino acid insertions in domain I of *P. lucens* AMA-1 are shown in yellow.

An alignment of the translated sequences revealed three additional amino acid residues (Glu-Phe-X) positioned near hydrophobic amino acids that line the hydrophobic trough of domain I (Additional file 8). The additional amino acids are located between residues 184-185 relative to *P. falciparum* AMA-1. Considering that it is unknown whether the tertiary structure of AMA-1 in avian *Plasmodium* species contains a hydrophobic trough, a 3D model of the full-length *P. gallinaceum* AMA-1 was generated. Tertiary structure-based analysis of the resulting 3D model was found to be satisfactory (LGscore = 3, MaxSub = 0.3). Conserved hydrophobic amino acid residues that line the hydrophobic trough, according to *P. falciparum* AMA-1 positions found by Bai *et al.* [75], were highlighted in green. All highlighted residues (111, 125, 132, 144, 299, 313 relative to *P. gallinaceum* AMA-1) also appeared to reside in a small yet extended pocket of *P. gallinaceum* AMA-1 (Figure 5A), with the exception of residues 193 and 194. 3D models for the AMA-1 domain I of *P. lucens* and *P. falciparum* were also generated to visually compare intraspecific polymorphisms (Figure 5B).

To determine whether these polymorphisms and additional amino acids are present among other avian *Plasmodium* species, domain I of *ama*-*1* from 28 *P. homopolare* (a newly identified host-generalist), three *P. megaglobularis* (host-generalist), three *Plasmodium* lineage spp. PV16, and two *P. globularis* (host-specialist) isolates [14, 54] were sequenced. Between-species divergence (*K*) ranged 0.062-0.349 (Additional file 4), approximately two to three-fold greater than K calculated for *cytochrome b* (0.030-0.103). Attempts to sequence domain I from other avian species, including *Plasmodium relictum* (GRW11 and SGS1) isolates were unsuccessful, likely due to low parasitaemia or parasite genomic DNA concentrations. Interestingly, no intraspecific polymorphisms were observed. However, additional amino acids in domain I were present in all field-caught avian *Plasmodium* species included in this study. Similar to *P. lucens*, domain I of *P. megaglobularis*, lineage PV16, and *P. globularis* AMA-1 sequences contain three additional amino acids (Glu-Phe-X) between residues 184-185, whereas the domain I of *P. homopolare* AMA-1 contains two amino acid (Arg-Asp) insertions between residues 187-188 (Additional file 8). All additional amino acids observed were located within or near hydrophobic amino acids that line the hydrophobic pocket.

### Comparison of *Plasmodium lucens ama-1*and *Plasmodium falciparum ama-1*sequences

Forty-nine *P. falciparum ama*-*1* sequences were compared with 51 *P. lucens ama*-*1* sequences in an alignment covering the entire domain I region. There were a total of 120 fixed nucleotide differences between the species. Of these differences, 33% (39) were synonymous and 67% were (81) non-synonymous. There were a total of 121 polymorphic sites within-species, of which 22% (27) sites were synonymous and 78% (94) were non-synonymous. A McDonald-Kreitman test with domain I sequences detected significant departure from neutrality in the *P. lucens ama*-*1* domain I region (Neutrality Index (NI) = 1.676, P = 0.07; NI with Jukes and Cantor correction = 2.035, P = 0.009), suggesting that polymorphisms at the domain I are maintained under positive diversifying selection.

A *d*_N_/*d*_S_ analysis of the entire domain did not detect significant differences between non-synonymous and synonymous changes. To determine if regions with high *d*_N_/*d*_S_ ratios are under selection, a sliding-window analysis (90 bp with a step size of three bases) of *d*_N_/*d*_S_ was conducted. Significant difference between *d*_N_ and *d*_S_ was detected throughout domain I (at the midpoint nt position of region 543-650, *d*_N_/*d*_S_ = 3.51, P < 0.0005; mid point nt position of region 609-698, *d*_N_/*d*_S_ = 1.90, P < 0.05; mid point nt position of region 645-735, *d*_N_/*d*_S_ = 1.97, P < 0.05; mid point nt position of region 657-746, *d*_N_/*d*_S_ = 1.66, P = 0.05; mid point nt position of region 669-758, *d*_N_/*d*_S_ = 2.01, P < 0.05) (Figure 4B), suggesting that these regions are under positive diversifying selection.

**Figure 6:**
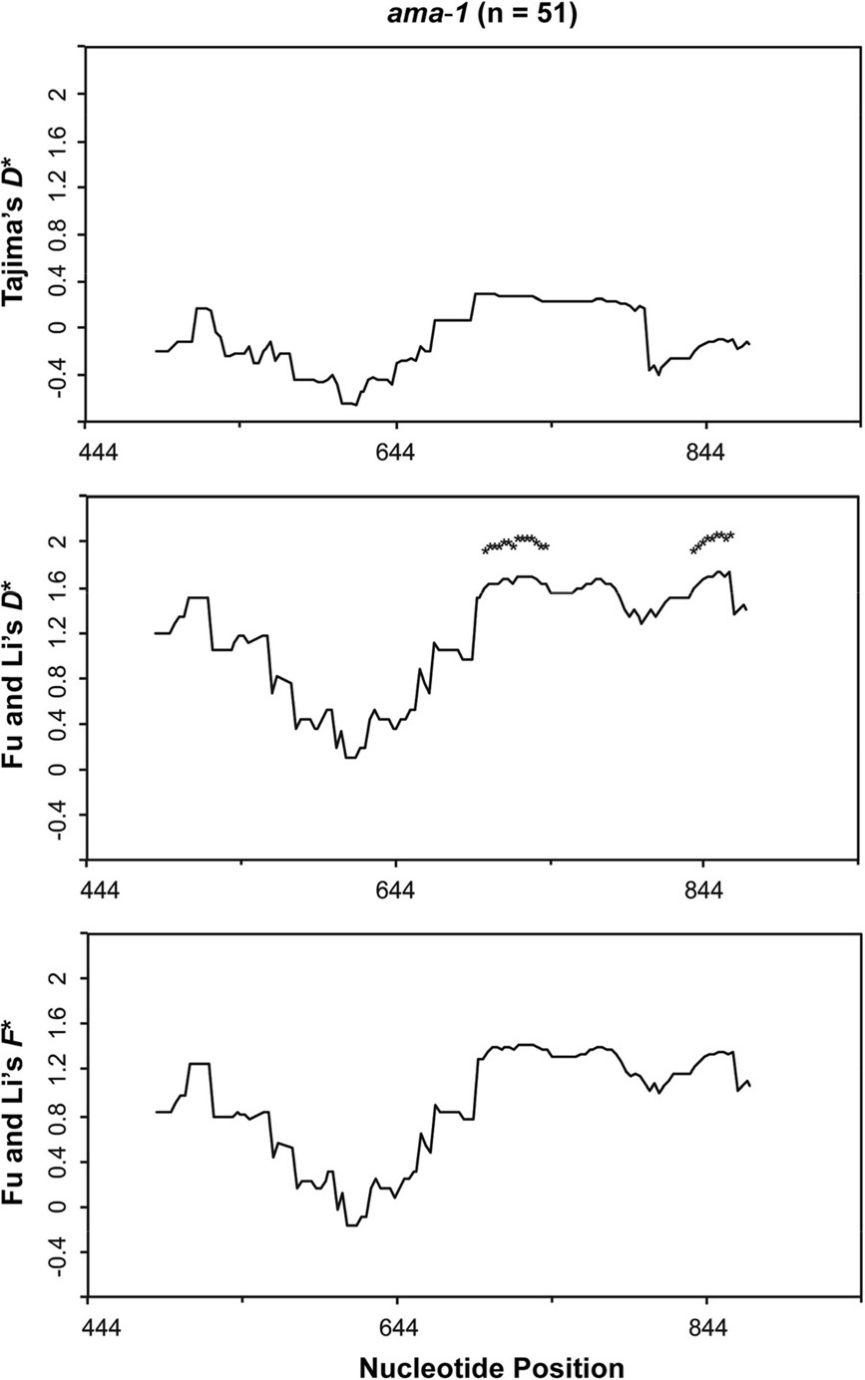
**Sliding window plot of Tajima’s** ***D*** **, Fu and Li’s** ***D*** * and ***F*** * tests for domain I of ***Plasmodium lucens ama*** **-** ***1*** **.** The window length is 90 bp with a step size of 3 bp. Asterisks indicate regions where significant departure from neutrality was observed. n represents the number of *P. lucens* sequences analysed.

No significant departure from the neutral expectation was detected using Tajima’s *D* (*D* = -0.12236, P > 0.10). Similar results were obtained for the Fu and Li D* (1.32144, P > 0.10), whereas the F* value of 1.79767 was significant with P < 0.02. Sliding-window analysis with a window of 30 bp and a step size of nine bases did not detect significantly low or high statistics values for all neutrality tests along the domain I region of *ama*-*1*. To ensure that the true level of variation is not obscured from choosing too small of a window, the sliding-window analysis with a window of 90 bp and a step size of three bases was performed. Significantly high F* values were observed between 658-786 bp and 718-831 bp (Figure 6). These results of the population-based methods also support the findings from comparing *d*_N_ to *d*_S_, and provide additional evidence that the domain I region of *P. lucens ama*-*1* is under positive diversifying selection.

## Discussion

Assessing the genetic diversity and selection in erythrocyte invasion genes of avian malaria parasites may provide insight on the population and the transmission dynamics of malaria. Higher rates of malaria transmission are expected to occur in areas with high parasite diversity [76]. This prediction is supported by the high levels of diversity in nuclear genes from malaria parasites found in avian hosts of Hawaii, including the thrombospondin-related anonymous protein (*trap*) gene, which encodes a protein involved in immuno-evasion and erythrocyte invasion [77]. Although *trap* evolves under positive selection in human malaria parasites, no evidence for positive selection was found in *trap* of avian malaria parasites [78]. Here, for the first time, evidence of positive selection as a driving force in the evolution and diversification of an erythrocyte invasion gene (*ama*-*1*) in avian malaria parasites is provided. These results show that *ama*-*1* is a useful nuclear marker for investigating the adaptive evolution of avian malaria parasite populations. In addition, these results suggest that the role *ama*-*1* plays in erythrocyte invasion is evolutionarily conserved across avian *Plasmodium* species.

Avian *Plasmodium ama*-*1* is relatively conserved in comparison with orthologous genes of mammalian *Plasmodium* species. The conservation of the hydrophobic trough in *P. gallinaceum* AMA1 underscores the functional importance of AMA-1 in avian *Plasmodium* parasites. The hydrophobic trough of AMA-1 binds to the RON complex via AMA-1-RON2 interactions [73, 79]. These interactions form the invasion machinery required to mediate erythrocyte invasion [80, 81]. Moreover, the identification of *P. gallinaceum* RON2 and the conservation of the RON2 helices involved in AMA-1 binding further supports the notion that this junction-dependent invasion process is evolutionarily conserved in avian *Plasmodium* parasites. Future studies will be important to investigate the signatures of selection within regions of *P. gallinaceum* RON2, and to further the understanding of junction-dependent invasion processes in avian *Plasmodium* parasites.

Although this process is not specific to a particular host cell type, the binding of domain III to the erythrocyte membrane protein Kx suggests that AMA-1 may be involved in host-specificity [82]. Interestingly, domain III is not well conserved in other genera belonging to Apicomplexa [82], suggesting that the functions of this domain are unique to *Plasmodium*. More importantly, domain III of *P. falciparum* AMA-1 is antigenic and elicits growth-inhibiting antibodies [83]. Domain III contains two conserved immunodominant epitopes. The first conserved epitope is located at position 459-464, whereas the second is located at position 467-475. Domain III of *P. gallinaceum* AMA-1 lacks four amino acid residues within the second immunodominant epitope (between position 468-473 with respect to *P. falciparum* AMA-1). These differences may also be present in other avian *Plasmodium* species and possibly result in a lack of or escape from inhibitory antibodies directed against the second immunodominant epitope. It is thus tempting to speculate that the observed differences in the domain III region of *P. gallinaceum* AMA-1 may contribute to the diverse host range of avian *Plasmodium* parasites. Unfortunately, efforts to PCR amplify and assess the domain III regions in *ama*-*1* of other avian *Plasmodium* parasites were unsuccessful.

In spite of this, several avian *Plasmodium* species exhibited genetic diversity and contained additional amino acids present within the domain I region of AMA-1. *Plasmodium* isolates from African birds contained three additional amino acids in domain I, whereas *P. homopolare* isolates from California contained two additional amino acids. Surprisingly, domain I of *P. homopolare ama*-*1* was highly conserved with no genetic diversity found between 28 different isolates. One possibility for this finding is that domain I of *P. homopolare ama*-*1* is subject to negative selection, as negative selection has been shown on the rhoptry-associated protein 1 (RAP-1), which is also involved in erythrocyte invasion [84]. However, this possibility is difficult to investigate considering the lack of diversity in *P. homopolare ama*-*1*. Alternatively, the lack of diversity may result from a recent demographic sweep (bottle-neck) or host switching events that lead to recent population expansions [85, 86]. The latter is favoured, as *P. homopolare* was found in five different families representing nine bird species [54]. Therefore, any reciprocal selection acting on *P. homopolare ama*-*1* by host protective immune responses may be weak relative to the selection imposed on a host specialist by a single host (e.g., the Olive Sunbird and *P. lucens*). A lack of diversity was also found in the host generalist *P. megaglobularis*. However, more robust sampling and sequencing of parasites is required as the true diversity may be underestimated due to low sample sizes. These results are in stark contrast to the level of genetic diversity found in *P. lucens ama*-*1*.

These findings provide significant evidence that polymorphisms in *P. lucens ama*-*1* are maintained by positive selection. The patterns of genetic diversity across domain I of *P. lucens ama*-*1* are consistent with studies on mammalian *Plasmodium* species [72, 74, 87, 88]. Likewise, sliding window plots of π and Fu and Li’s F* value indicate that the region corresponding to a natural T cell epitope in domain I is highly polymorphic and is under positive selection in *P. lucens ama*-*1*, which is also in agreement with earlier studies [42, 89]. This observation suggests that avian *Plasmodium* AMA-1 may induce T cell responses in infected bird hosts and has implications for studying immune responses in bird populations that are naturally exposed to malaria parasites. In addition, humoral immune responses against AMA-1 in bird populations may also provide valuable information for immuno-epidemiologic studies. Such studies can be particularly important for terrestrial ecosystems that are sensitive to losses in native bird populations, especially since native birds play essential roles in many terrestrial ecosystems [90, 91]. Therefore, there is a great need to understand host-parasite interaction and its relationship to host immune responses in avian *Plasmodium* parasites.

## Conclusions

Ultimately, these findings provide insight into the erythrocyte invasion process of avian *Plasmodium*, are the first evidence of host-driven selection in an avian *Plasmodium* species, and demonstrate the substantial applications of the *P. gallinaceum* transcriptome. The *P. gallinaceum* transcriptome dataset represents a major public genomic resource that will serve to progress research on the functional genomics and evolution of *Plasmodium*. Further analyses of invasion and immuno-evasion-related genes could reveal additional nuclear markers for phylogenetic applications, as these genes may exhibit high levels of diversity [21]. Identifying additional markers has been difficult as the majority of sequenced *Plasmodium* genomes are from mammalian *Plasmodium* species; therefore, primer development is often facilitated using mammalian *Plasmodium* species sequence data and the resulting primers may not be suitable for PCR amplification of non-mammalian *Plasmodium* species DNA. However, with the recent sequencing and availability of the *P. relictum* transcriptome [21] and the addition of the *P. gallinaceum* transcriptome, significant progress towards adding reliable markers for more thorough phylogenetic and evolutionary studies of *Plasmodium* or closely related genera is expected.

## Electronic supplementary material

Additional file 1: **Host and parasite species locality.** The table shows the source of isolates used to analyse sequence diversity. (DOCX 47 KB)

Additional file 2: **GenBank Accession numbers for the parasite taxa.** GenBank Accession numbers for the parasite taxa used in this study are provided. (DOCX 68 KB)

Additional file 3: **Genbank accession numbers for the** ***ama***-***1*** **sequences.** The table shows Genbank accession numbers for the *ama*-*1* sequences obtained from *Plasmodium* field isolates used in this study. (DOCX 82 KB)

Additional file 4: **Between-species divergence.** The table shows between-species divergence (K) calculated using Jukes and Cantor correction. (DOCX 47 KB)

Additional file 5: **Genetic distances and amino acid similarity.** The table shows genetic distances computed from DNA sequences and amino acid similarity between *Plasmodium gallinaceum* and *Plasmodium falciparum*. (DOCX 41 KB)

Additional file 6: **The size distribution for** ***Plasmodium gallinaceum*** **transcripts.** The figure shows the size distribution for *Plasmodium gallinaceum* transcripts that show homology to *Plasmodium falciparum* transcripts. (DOCX 106 KB)

Additional file 7: **Phylogenetic and MDS analysis of** ***Plasmodium*** **parasites.** An *ama-1* phylogeny and MDS analysis shows clustering of avian malaria parasites according to host species. (DOCX 1 MB)

Additional file 8: **AMA1- domain I amino acid sequence alignment.** The figure shows aligned amino acid sequences with the AMA-1 domain I of *Plasmodium falciparum*, *Plasmodium lucens*, *Plasmodium megaglobularis*, *Plasmodium globularis*, *Plasmodium* lineage spp. PV16, and *Plasmodium homopolare*. (DOCX 13 MB)
