# Supplementary material for: Transcriptome sequencing and analysis of Plasmodium gallinaceum reveals polymorphisms and selection on the apical membrane antigen-1
Source: Malar J. 2014 Sep 26;13:382. doi: 10.1186/1475-2875-13-382 (PMC4182871; doi:10.1186/1475-2875-13-382)
Supplement: Supplementary file 1 — Additional file 1: Host and parasite species locality. The table shows the source of isolates used to analyse sequence diversity. (DOCX 47 KB) [file 12936_2014_3545_MOESM1_ESM.docx]

| Plasmodium Species | Host species | N | Locality |
| --- | --- | --- | --- |
| *P. lucens* | Olive sunbird, *Cyanomitra olivacea* | 51 | Cameroon |
| *P. homopolare* | Common yellowthroat, *Geothlypis trichas* | 1 | California |
|  | Song sparrow, *Melospiza melodia* | 15 |  |
|  | Spotted towhee, *Pipilo maculatus* | 12 |  |
| *P. globularis* | Yellow-whiskered greenbul, *Andropadus latirostris* | 2 | Ghana |
| *P. megaglobularis* | Olive-bellied sunbird, *Cinnyris chloropygius* | 3 | Cameroon |
| PV16 | Olive sunbird, *Cyanomitra olivacea* | 3 | Cameroon |

**Table S1**

Source of isolates used to analyse sequence diversity.
