# Supplementary material for: Transcriptome sequencing and analysis of Plasmodium gallinaceum reveals polymorphisms and selection on the apical membrane antigen-1
Source: Malar J. 2014 Sep 26;13:382. doi: 10.1186/1475-2875-13-382 (PMC4182871; doi:10.1186/1475-2875-13-382)
Supplement: Supplementary file 2 — Additional file 2: GenBank Accession numbers for the parasite taxa. GenBank Accession numbers for the parasite taxa used in this study are provided. (DOCX 68 KB) [file 12936_2014_3545_MOESM2_ESM.docx]

| Plasmodium Species | Accession Nos/Gene IDs |
| --- | --- |
| *P. falciparum* | XM001347979, XM001350714, PF3D70920000, XM_001348670, PF3D71452000 |
| *P. knowlesi* | AF298218, XM002262406, PKH071720, XM002260470, M |
| *P. vivax* | AF063138, XM_001613842, XM001613489, XM001615779, PVX117880 |
| *P. berghei* | U45969, XM673698, PBANKA082090, XM675246, PBANKA131570 |
| *P. yoelii yoelii* | XM724270, XM722931, PYYM0823900, PYYM1320300, PY17X1319500 |
| *P. chabaudi* | U49743, XM739020, PCHAS082120, PCHAS132280, PCHAS131900 |
| *P. cynomolgi* | XM004222507, XM004225083, XM004220500, XM004223926, PCYB126310 |
| *P. reichenowi* | AJ252087, M, M, M |
| *P. gallinaceum* | KJ722597, KJ722601, KJ722599, KJ722598, KJ722600 |

**Table S2**

GenBank Accession numbers for the parasite taxa used in the study. GenBank Accession numbers for the parasite taxa are provided in the gene order: *ama*-*1*, *SHMT* *ELO3*, *CCp2*, and *RON2*. M, missing gene sequence data.
