# Supplementary material for: Transcriptome sequencing and analysis of Plasmodium gallinaceum reveals polymorphisms and selection on the apical membrane antigen-1
Source: Malar J. 2014 Sep 26;13:382. doi: 10.1186/1475-2875-13-382 (PMC4182871; doi:10.1186/1475-2875-13-382)
Supplement: Supplementary file 3 — Additional file 3: Genbank accession numbers for the ama-1 sequences. The table shows Genbank accession numbers for the ama-1 sequences obtained from Plasmodium field isolates used in this study. (DOCX 82 KB) [file 12936_2014_3545_MOESM3_ESM.docx]

| Plasmodium ID | Accession Nos |
| --- | --- |
| *P. lucens 250923* | KJ722542 |
| *P. lucens 250930* | KJ722543 |
| *P. lucens 250988* | KJ722544 |
| *P. lucens 251070* | KJ722545 |
| *P. lucens 251074* | KJ722546 |
| *P. lucens 251081* | KJ722547 |
| *P. lucens 251473* | KJ722548 |
| *P. lucens 251937* | KJ722549 |
| *P. lucens 251776* | KJ722550 |
| *P. lucens 251791* | KJ722551 |
| *P. lucens 251823* | KJ722552 |
| *P. lucens 251995* | KJ722553 |
| *P. lucens 271698* | KJ722554 |
| *P. lucens 271752* | KJ722555 |
| *P. lucens 271959* | KJ722556 |
| *P. lucens 271961* | KJ722557 |
| *P. lucens 271962* | KJ722558 |
| *P. lucens 271963* | KJ722559 |
| *P. lucens 271967* | KJ722560 |
| *P. lucens 271968* | KJ722561 |
| *P. lucens 271978* | KJ722562 |
| *P. lucens 271980* | KJ722563 |
| *P. lucens 271982* | KJ722564 |
| *P. lucens 271983* | KJ722565 |
| *P. lucens 271984* | KJ722566 |
| *P. lucens 271994* | KJ722567 |
| *P. lucens 271996* | KJ722568 |
| *P. lucens 272544* | KJ722569 |
| *P. lucens 272556* | KJ722570 |
| *P. lucens 272562* | KJ722571 |
| *P. lucens 272566* | KJ722572 |
| *P. lucens 272575* | KJ722573 |
| *P. lucens 272586* | KJ722574 |
| *P. lucens 272598* | KJ722575 |
| *P. lucens 272608* | KJ722576 |
| *P. lucens 272625* | KJ722577 |
| *P. lucens 272635* | KJ722578 |
| *P. lucens 272653* | KJ722579 |
| *P. lucens 272680* | KJ722580 |
| *P. lucens 272686* | KJ722581 |
| *P. lucens 272687* | KJ722582 |
| (Table S3 continued…) |  |
| *P. lucens 272690* | KJ722583 |
| *P. lucens 272696* | KJ722584 |
| *P. lucens 272701* | KJ722585 |
| *P. lucens 272703* | KJ722586 |
| *P. lucens 272751* | KJ722587 |
| *P. lucens 272756* | KJ722588 |
| *P. lucens 250073* | KJ722589 |
| *P. lucens 250250* | KJ722590 |
| *P. lucens 250323* | KJ722591 |
| *P. lucens 251120* | KJ722592 |
| *P. megaglobularis 909153...* KJ722593 | |
| *P. globularis 271155* | KJ722594 |
| *PV16 251183* | KJ722595 |
| *P. homopolare CA52* | KJ722596 |

**Table S3**

Genbank accession numbers for the *ama*-*1* sequences obtained from *Plasmodium* field isolates used in this study.
