# Supplementary material for: Transcriptome sequencing and analysis of Plasmodium gallinaceum reveals polymorphisms and selection on the apical membrane antigen-1
Source: Malar J. 2014 Sep 26;13:382. doi: 10.1186/1475-2875-13-382 (PMC4182871; doi:10.1186/1475-2875-13-382)
Supplement: Supplementary file 4 — Additional file 4: Between-species divergence. The table shows between-species divergence (K) calculated using Jukes and Cantor correction. (DOCX 47 KB) [file 12936_2014_3545_MOESM4_ESM.docx]

|  | *P. gal* | *P. luc* | *P. hom* | *P. meg* | *P. glo* | PV16 |
| --- | --- | --- | --- | --- | --- | --- |
| *P. gal* | - | **0.0697** | **0.0985** | **0.0580** | **0.1034** | **0.0768** |
| *P. luc* | 0.2993 | - | **0.0791** | **0.0580** | **0.0791** | **0.0301** |
| *P. hom* | 0.2832 | 0.2953 | - | **0.0839** | **0.0397** | **0.0839** |
| *P. meg* | 0.2697 | 0.1443 | 0.3057 | - | **0.0839** | **0.0487** |
| *P. glo* | 0.3181 | 0.3266 | 0.2069 | 0.3532 | - | **0.0839** |
| PV16 | 0.2984 | 0.0609 | 0.3122 | 0.1641 | 0.3429 | - |

**Table S4**

Between-species divergence (K) calculated using Jukes and Cantor correction. Bold numbers represent K values for *cytochrome b*. Unbolded numbers represent K values for domain I of *ama*-*1.*
