# Supplementary material for: Transcriptome sequencing and analysis of Plasmodium gallinaceum reveals polymorphisms and selection on the apical membrane antigen-1
Source: Malar J. 2014 Sep 26;13:382. doi: 10.1186/1475-2875-13-382 (PMC4182871; doi:10.1186/1475-2875-13-382)
Supplement: Supplementary file 5 — Additional file 5: Genetic distances and amino acid similarity. The table shows genetic distances computed from DNA sequences and amino acid similarity between Plasmodium gallinaceum and Plasmodium falciparum. (DOCX 41 KB) [file 12936_2014_3545_MOESM5_ESM.docx]

| **Gene** | **p-distance** | **Jukes-Cantor** | **Amino acid similarity (%)** |
| --- | --- | --- | --- |
| *SHMT* | 0.21 | 0.24 | 84 |
| *ELO3* | 0.25 | 0.3 | 79 |
| *CCp2* | 0.23 | 0.27 | 74 |
| *ama-1* | 0.32 | 0.41 | 55 |
| *RON2* | 0.28 | 0.36 | 71 |

**Table S5**

Genetic distances computed from DNA sequences and amino acid similarity between *Plasmodium gallinaceum* and *Plasmodium falciparum*.
