# Supplementary material for: Transcriptome sequencing and analysis of Plasmodium gallinaceum reveals polymorphisms and selection on the apical membrane antigen-1
Source: Malar J. 2014 Sep 26;13:382. doi: 10.1186/1475-2875-13-382 (PMC4182871; doi:10.1186/1475-2875-13-382)
Supplement: Supplementary file 6 — Additional file 6: The size distribution for Plasmodium gallinaceum transcripts. The figure shows the size distribution for Plasmodium gallinaceum transcripts that show homology to Plasmodium falciparum transcripts. (DOCX 106 KB) [file 12936_2014_3545_MOESM6_ESM.docx]

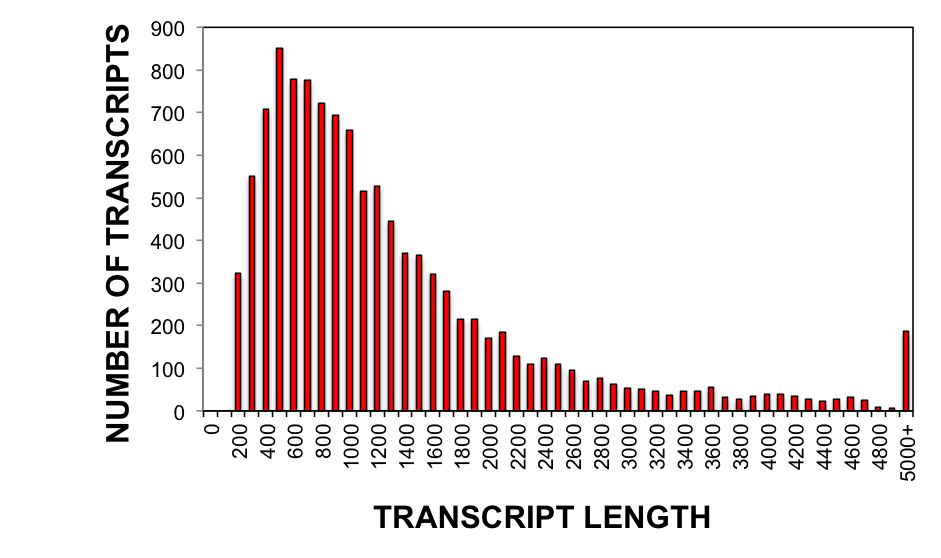


**Figure S1**

The size distribution for *Plasmodium gallinaceum* transcripts that show homology to *Plasmodium falciparum* transcripts. All *P. gallinaceum* coding sequences (CDS) were searched against the *P. falciparum* transcriptome found in Plasmodiumdb.
