# Supplementary material for: Transcriptome sequencing and analysis of Plasmodium gallinaceum reveals polymorphisms and selection on the apical membrane antigen-1
Source: Malar J. 2014 Sep 26;13:382. doi: 10.1186/1475-2875-13-382 (PMC4182871; doi:10.1186/1475-2875-13-382)
Supplement: Supplementary file 7 — Additional file 7: Phylogenetic and MDS analysis of Plasmodium parasites. An ama-1 phylogeny and MDS analysis shows clustering of avian malaria parasites according to host species. (DOCX 1 MB) [file 12936_2014_3545_MOESM7_ESM.docx]

****A

B

**Figure S2**

Phylogenetic and MDS analysis of *Plasmodium* parasites based on the domain I region of *ama*-*1*. The phylogeny based on domain I of *ama*-*1* A) only shows bootstrap values above 70. Clades are colored according to parasite host’s; avian malaria parasites (green), primate malaria parasites (blue), rodent malaria parasites (red). The field isolate sequences used in this phylogeny are identified with arbitrary ID codes. Accession numbers identify the *Pf ama*-*1* sequences. B) PCA plots represented by MDS are shown for domain I of *ama*-*1*. For accession numbers see Table S4.
