# Supplementary material for: Transcriptome sequencing and analysis of Plasmodium gallinaceum reveals polymorphisms and selection on the apical membrane antigen-1
Source: Malar J. 2014 Sep 26;13:382. doi: 10.1186/1475-2875-13-382 (PMC4182871; doi:10.1186/1475-2875-13-382)
Supplement: Supplementary file 8 — Additional file 8: AMA1- domain I amino acid sequence alignment. The figure shows aligned amino acid sequences with the AMA-1 domain I of Plasmodium falciparum, Plasmodium lucens, Plasmodium megaglobularis, Plasmodium globularis, Plasmodium lineage spp. PV16, and Plasmodium homopolare. (DOCX 13 MB) [file 12936_2014_3545_MOESM8_ESM.docx]

**Figure S3**

Aligned amino acid sequences with the AMA-1 domain I of *Plasmodium falciparum*, *Plasmodium lucens*, *Plasmodium megaglobularis*, *Plasmodium globularis*, *Plasmodium* lineage spp. PV16, and *Plasmodium homopolare*. Dots represent identical amino acids. Conservation level at individual sequence positions are shown as bar plots at the bottom of the alignment. Amino acid insertions relative to *Plasmodium falciparum* AMA-1 amino acid sequences are boxed in red.
